# Evaluation of Apical Micro-leakage of Different Endodontic Sealers in the Presence and Absence of Moisture

**DOI:** 10.5681/joddd.2014.023

**Published:** 2014-09-17

**Authors:** Maryam Ehsani, Atena Dehghani, Farida Abesi, Soraya Khafri, Sara Ghadiri Dehkordi

**Affiliations:** ^1^Dental Materials Research Center, Babol University of Medical Sciences, Babol, Iran; ^2^Associate Professor, Department of Endodontics, Faculty of Dentistry, Babol University of Medical Sciences, Babol, Iran; ^3^Under-graduate student, Student Research Center, Faculty of Dentistry, Babol University of Medical Sciences, Babol, Iran; ^4^Assistant Professor, Department of Oral & Maxillofacial Radiology, Faculty of Dentistry, Babol University of Medical Sciences, Babol, Iran; ^5^Assistant Professor, Department of Social Medicine and Health, Faculty of Medicine, Babol University of Medical Science, Babol, Iran; ^6^Dentist, Private Practice, Babol, Iran

**Keywords:** Micro-leakage, moisture, root canal therapy, sealer

## Abstract

***Background and aims.*** With availability of hydrophilic sealers, drying of the canals before endodontic obturation is still a matter of debate. The aim of this study was to compare the apical micro-leakage of AH26, Excite DSC, MTA Fillapex, and ZOE sealers in dry and moist root canals.

***Materials and methods.*** This experimental study was performed on 90 extracted maxillary central incisors. Rotary files were used for preparation of the canals. Root canals were filled with a single gutta percha cone, using one of the four sealers, under dry and moist root canal conditions (10 teeth in each group). Orifices were sealed with glue wax and all root surfaces were covered with nail polish except the positive control group. After ten days in 100% humidity, teeth were placed in methylene blue, and then were cut in longitudinal axis. Blue color permeability was measured by stereomicroscope in micrometers. Data were analyzed by t-test, ANOVA and Scheffe post hoc test using SPSS V.18 software at P < 0.05.

***Results.*** Mean apical micro-leakage was significantly lower in the dry groups (P < 0.001). Minimum and maximum micro-leakage was seen in AH26 and ZOE, respectively. MTA Fillapex did not exhibit a significant difference in apical micro-leakage between dry and moist conditions (P > 0.05). Apical micro-leakage was significantly higher in the Excite DSC groups (P < 0.001).

***Conclusion.*** AH26 provided the least apical micro-leakage under dry conditions while ZOE had the highest micro-leakage under moist conditions. MTA Fillapex provided acceptable apical seal regardless of moisture.

## Introduction

Endodontic sealers are commonly used with gutta percha to reach an optimal apical seal.^1^ Improper apical seal has been reported as the most common cause of root treatment failure.^2,3^An effective endodontic seal blocks communication between apical foramen and surrounding pre-apical tissues.^4^ It is generally recommended to dry the root canal before obturation, as this increases the sealers adherence to the dentin walls of the canal and the filling material.^1-3^ Moisture may prevent sealer setting by increasing or reducing its working or setting time,^4-6^ and may interface the entrance of the sealer into the dentinal tubules.^7^ Excite DSC is a dual-cure sealer with adhesive properties to dentin and enamel, and solubility in alcohol and a few study were conducted about it as a root canal sealer.^8,10^ MTA Fillapex, a mineral trioxide aggregate (MTA) based sealer, composed of resins (salicylate, diluting, natural), radiopaque bismuth, nanoparticulated silica, MTA, and pigments. To date, only scant knowledge is available with regard to its adhesive properties.^11^ It has the ISO 6786 root canal sealers standard, in addition having all the beneficial properties of MTA.^12^

 Several studies have been done about the sealing ability of different sealers,^13-15^ and different levels of residual moisture in the root canal have been shown to alter the sealing properties of conventional and resin-based sealers.^11^ However, there are few studies about apical micro-leakage using Excite DSC and MTA Fillapex sealers in comparison to other widely used sealers, or about the effect of the moisture on apical microleakage of the sealers.^4-7,16-18^ Many studies have proposed that apical microleakage occurs due to the prior existence of blood and moisture in canal during obturation, but recent studies have claimed that remnant moisture does not alter the mean value of microleakage.^18^ Based on these observations, the present study was designed to compare the apical seal of AH26, Excite DSC, MTA Fillapex and ZOE sealers in the presence and absence of remaining moisture within the canal. 

## Materials and Methods

 This experimental study included 90 extracted teeth collected from dental clinics in the city of Babol, North of Iran. Inclusion criteria: Maxillary central incisor; no coronal restoration or decay below the cemento enamel junction (CEJ); complete root formation without signs of internal or external resorption; straight cone-shaped root without curvature in the apical third; no fracture or crack in the root; no calcification in the root canal. Exclusion criteria: K files #10 and #15 not passing beyond 11 mm from CEJ into the root canal.^5,10,16^

###  Preparation of the Teeth

 The collected teeth were cleaned immediately after extraction by removing all attached hard and soft tissues and immersing in 1000 ml of 5.25% sodium hypochlorite (Golrang Co., Tehran, Iran) for 24 hours. Then the teeth were stored in the container with lid containing 0.9% sterile saline (Iran transfusion product Co., Tehran, Iran) at room temperature until further processing. Before starting the study, teeth crown were cut near the CEJ using a diamond disc (Tizkavan, Tehran, Iran) and a high-speed handpiece (NSK, Tokyo, Japan) with water coolant perpendicular to the long axis, to achieve a length of 12 mm for all samples, measured by a digital caliper (Goanjigo SR 44, China). All prepared teeth were again held in 0.9% sterile saline at room temperature until the test time. 

 For root canal preparation, micro motor system (C-Smart; NSK, Tokyo, Japan) and rotary files (Mtwo; VDW, GmbH, Germany) with single length technique were used. The system settings for all teeth were as follows: speed ​​= 375 rpm, torque = 20 mn.m, program 2. The root canal was dried with #30 paper points. To confirm drying of the canal, five consecutive #30 paper points were placed in the canal for five seconds and had to remain dry. 

 The teeth were randomly divided into one control and eight test groups, each containing ten specimens. 

### Application of Sealers

 The four tested sealers were the following: ZOE sealer (Golchai Co. Iran), AH26 sealer (Dentsply, Detrey, Germany), MTA Fillapex sealer (Angelus Indústria de Produtos Odontológicos S/A, Londrina, Brazil), and Excite DSC sealer (Ivocolar Vivadent, Germany). All sealers were prepared according to the instructions of the manufacturers. Upon preparation, the sealer was placed in the syringe. The syringe needle was placed in the canal at a length of 11 mm from the CEJ and the sealer was infused in the canal. In order to ensure the complete filling of the apical region of the canal, visible sealer extrusion from the apical foramen was noted. The syringe was slowly drawn back, allowing the sealer to fill the canal until the sealer discharged out of the orifice. Then using a forceps, a standard #30 gutta-percha cones (GAPADENT Co., China) was placed in the canal at the length of 11 mm. In the Excite DSC sealer group, the specimens were light-cured for 40 seconds. 

 The same procedure was repeated for another four groups in moist condition. For canal wetting, with an insulin syringe needle (Helal Medical Equipment Co., Tehran, Iran) 0.02 ml saline was poured into the canal. 

 Five teeth as a positive control and five teeth as negative controls remained unfilled after canal preparation. 

 In all teeth, the root canal orifices were sealed with glue wax (Golchai, Tehran, Iran). Teeth were then covered with two layers of nail polish except for the apical 1 mm of the root tip. Apical foramina of the teeth in negative controls were also covered with glue wax. The specimens were stored in an incubator at the controlled temperature of 37°C with 100% humidity for ten days in order to complete the sealer setting reaction. Then, the samples were immersed in methylene blue for 3 days. 

### Measurement of Micro-leakage

**Figure 1.**
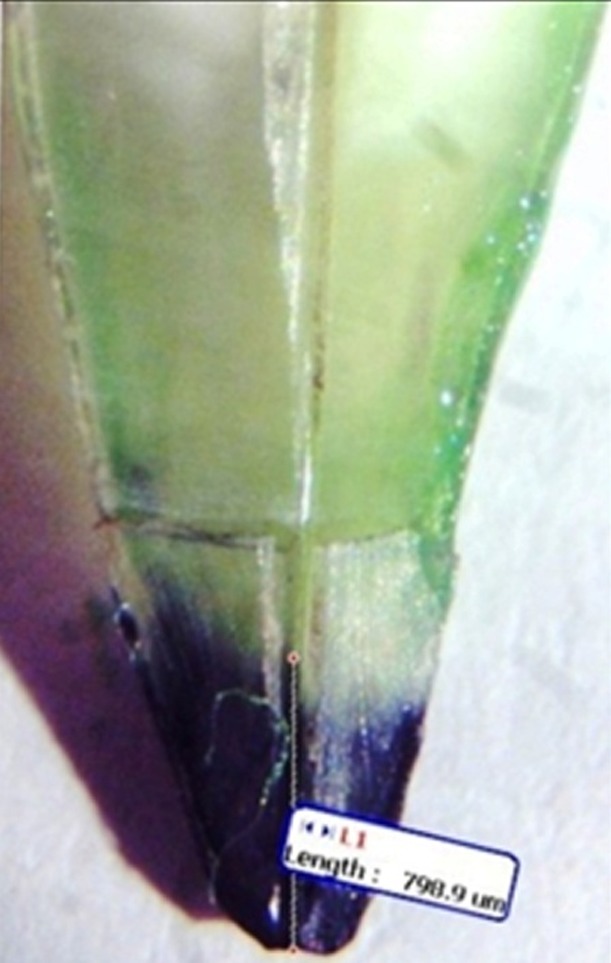
Measurement of dye penetration to the long axis of the tooth.

The teeth were sectioned using a disc on a high-speed handpiece (Kavo, GmbH, Germany) along the longitudinal axis close to the center of the canal and then were split. To measure dye penetration, a stereomicroscope (SMZ1, Tokyo, Japan) was used with ×2 magnification. The amount of leakage was measured from the apex to the highest amount of dye penetration in micrometers using computer software (Motic Image version 2.20) on the image captured by a digital camera (Moticam 2000, Tokyo, Japan) mounted on the stereomicroscope (Figure 1).

### Statistical Analysis

 The data were analyzed by t-test, ANOVA and Scheffe using SPSS v.18 statistical software. P value < 0.05 was considered statistically significant. 

## Results

 Mean total apical micro-leakage of all study sealers was 324.97 ± 149.37 µm (dry canal groups, 299.39 ± 143.63 µm; moist canal groups, 350.56 ± 152.38 µm). The lowest rate of apical micro-leakage was seen in AH26 sealer (138.50 ± 34.62 µm) and the highest apical micro-leakage was related to ZOE sealer (522.29 ± 61.13 µm). AH26 sealer had the lowest rate of apical micro-leakage in dry and wet canals (Table 1). 

**Table 1:** Comparison of mean apical micro-leakage (in micrometer) with four sealers evaluated under dry and moist conditions

Group	AH26	Excite	MTA Fillapex	ZOE	P-value	Total
Dry	116.83± 26.42^a^	256.47± 27.53^b^	331.26 ± 68.96^c^	493.00 ± 36.48^d^	<0.001	299.39 ± 143.63
Moist	160.17 ± 28.8^a^	314.83 ± 21.77^b^	375.65 ± 86.13^b^	551.59 ± 68.19^c^	<0.001	350.56 ± 152.38
P-value	0.02	<0.001	0.22	0.03	—	—
Total	138.50 ± 34.62	285.65 ± 38.46	353.43 ± 79.28	522.29 ± 61.13	—	—
Different letters in each row show statistical significant differences between groups at ? = 0.05.						

 ANOVA test showed statistically significant differences in the rate of apical micro-leakage between the studied sealers (P < 0.001; Table 1). Mean apical micro-leakage in all sealers was significantly lower on dry canal conditions compared with that in moist canal conditions, except for MTA Fillapex sealer which had no significant difference in micro-leakage rate between the two conditions (P = 0.22). There are statistically significant differences between different sealers in dry or moist conditions. Also, Scheffe multiple comparisons test showed that among the moist canal groups, there was no significant difference between MTA Fillapex and Excite DSC (P = 0.156; Table 1). Positive control group had dye penetration rate of 100% and negative control group had no dye penetration. 

## Discussion

 According the results of the present study, the lowest rate of micro-leakage was seen in AH26 group in dry and wet conditions, and the highest rate was seen in ZOE group. Also in MTA Fillapex group, there was no statistically significant difference in apical micro-leakage in dry and moist conditions. MTA Fillapex has been demonstrated previously to have a better apical seal compared to AH26.^8^ However, in the present study AH26 had the lowest rate of apical micro-leakage in the presence and absence of moisture. The individual difference in manipulation and application of sealers can be a reason for this difference. Further, the amount of moisture within the root canal may be a confounding factor that affects the apical micro-leakage. 

 The quality of adhesion between root canal dentin and sealers may also be affected by the moisture condition of the root canals before filling procedures. Despite the fact that the perception of moisture may vary widely among clinicians, several manufacturers recommend that the root canals be maintained in a moist state to benefit from the hydrophilic properties of their sealers without providing exact clinical instructions for achieving the degree of residual moisture that is ideal for their products.^11^ Nagas et al^11^ found that the degree of residual moisture significantly affects the adhesion of root canal sealers to radicular dentin. For the tested sealers including AHplus and MTA Fillapex, it may be advantageous to leave canals slightly moist before filling.^11^ Roggendorf et al^6^ showed that moisture led to less microleakage for Apexit, RoekoSeal, and Tubli-Seal and higher values for AH Plus and Ketac-Endo.^6^ They stated that moisture may work as a lubricant for these sealers that allows a better attachment to the root canal wall, thus, a complete drying of the root canal dentin may have adversely affected linear dye penetration.^6^

 With present methodology, a single gutta-percha cone was used with the sealers. Khalilak et al^18^ compared Resilon and Epiphany sealer with gutta-percha and AH26 sealer and found no significant difference in apical micro-leakage in moist condition after one day; however, micro-leakage was significantly lower in AH26 group after 3 weeks, a finding which is in line with that of the present study. Testing under moist and dry conditions had demonstrated by that AH26 sealer had the lowest micro-leakage and that the type of moisture (blood or 5.25% hypochlorite) had no significant effect on the rate of sealer micro-leakage.^19^ Jin-Ah et al^20^ demonstrated that the moisture condition of root canals at the time of obturation and the type of sealer that was used had a significant effect on leakage and sealing ability. Thus drying procedure according to sealer types is a critical step and should not be missed in endodontic treatment. Low micro-leakage in AH26 group can be attributed to its chemical composition that even under moist conditions allows complete setting of the sealer. This notion, however, must be regarded cautiously as moist conditions in the present study influenced the chemical reaction of the sealer in a way that a significant difference was observed for apical micro-leakage of AH26 between dry and moist groups. Within the limitations of the present study, MTA Fillapex among all sealers was the only sealer with no significant difference in micro-leakage in the presence or absence of moist conditions, confirming the claims of its manufacturer. 

 In order to replicate the clinical situation more precisely, it is suggested that similar work be conducted under conditions where blood or serum is present in the tested root canals. Further research focusing on the effects of different percentages of root canal moisture on apical seal could yield useful findings. 

## Conclusion

 Mean micro-leakage in AH26 sealer group in both moist and dry conditions was significantly lower than that of other sealers. Maximum micro-leakage was seen in the ZOE group under moist conditions. Overall, moisture had a negative effect on the apical seal, except for MTA Fillapex sealer. 
